# *Elaeagnus glabra f. oxyphylla* Attenuates Scopolamine-Induced Learning and Memory Impairments in Mice by Improving Cholinergic Transmission via Activation of CREB/NGF Signaling

**DOI:** 10.3390/nu11061205

**Published:** 2019-05-28

**Authors:** Eunjin Sohn, Hye-Sun Lim, Yu Jin Kim, Bu-Yeo Kim, Joo-Hwan Kim, Soo-Jin Jeong

**Affiliations:** 1 Clinical Medicine Division, Korea Institute of Oriental Medicine, Daejeon 34054, Korea; qp1015@kiom.re.kr (H.-S.L.); jinjin0228@kiom.re.kr (Y.J.K.); buykim@kiom.re.kr (B.-Y.K.); 2 Department of Life Science, Gachon University, Seongnam, Kyonggi-do 21936, Korea; kimjh2009@gachon.ac.kr

**Keywords:** memory dysfunction, scopolamine, cholinergic system, nerve growth factor (NGF), cAMP response element-binding protein (CREB), Alzheimer’s disease

## Abstract

We aimed to investigate the therapeutic effects of an *Elaeagnus glabra f.* *oxyphylla* (EGFO) ethanol extract in mice with scopolamine-induced memory dysfunction. Fifty male mice were randomly divided into a normal control group, a scopolamine-treated group, a scopolamine and EGFO extract-treated group, and a scopolamine and tacrine-treated group. EGFO (50 or 100 mg/kg/day) was received for 21 days. Step-through passive avoidance and Y-maze tests were performed to examine the effects of treatment on learning and memory impairments. Acetylcholine (Ach) levels and acetylcholinesterase (AchE) activity were measured via an enzyme-linked immunosorbent assay (ELISA). Levels of choline acetyltransferase (ChAT), nerve growth factor (NGF), cAMP response element-binding protein (CREB), and apoptosis-related protein expression were determined via Western blot analysis. EGFO pretreatment significantly attenuated scopolamine-induced memory impairments, relative to findings observed in the scopolamine-treated group. Levels of cholinergic factors in the brain tissues were markedly attenuated in the scopolamine-treated group. EGFO treatment also attenuated neural apoptosis in scopolamine-treated mice by decreasing the expression of apoptosis-related proteins such as Bax, Bcl2, cleaved caspase-3, and TUNEL staining. These results suggest that EGFO improves memory and cognition in a mouse model of memory impairment by restoring cholinergic and anti-apoptotic activity, possibly via activation of CREB/NGF signaling.

## 1. Introduction

Memory impairment is among the characteristic features of Alzheimer’s disease (AD), a progressive neurodegenerative disorder caused by neuronal dysfunction and loss in the brain [1]. As the number of older adults in the population continues to increase, so does the incidence of AD, representing a significant public health concern [2,3]. The neuropathological changes associated with memory loss alter levels of cholinergic markers in the central nervous system (CNS), including acetylcholine (Ach) and choline acetyltransferase (ChAT), which are correlated with the severity of AD [4,5,6,7]. Ach released from the hippocampus and cortex plays crucial roles in attention, learning, and memory [8,9]. Pharmacological interventions for AD that target the cholinergic system [10] modulate neurogenic mechanisms via cAMP response element-binding protein (CREB) and/or brain-derived neurotrophic factor (BDNF)-related pathways [8]. Research has suggested that apoptosis-related signaling is involved with dysfunction of the central cholinergic system and oxidative stress in various memory disorders, including AD [11,12]. Scopolamine (SCO) impairs memory in rodents and humans, which interferes with the cholinergic system including Ach, leading to memory impairments [13,14]. Among the AD drugs approved by the U.S. Food and Drug Administration (FDA), tacrine, donepezil, and rivastigmine, such as cholinesterase inhibitors, may increase levels of Ach [15]. However, their efficacy is relatively low, and they are involved with adverse effects such as vomiting, diarrhea, and dizziness [16,17]. *Elaeagnus glabra f. oxyphylla* (EGFO), which is morphologically distinct from *E. glabra*, is a small tree with narrow leaves that is native to Korea [18]. Previous studies have highlighted the pharmacological effects of various members of the *Elaeagnus* family. For instance, *E. glabra* exerts antibacterial and anti-asthmatic effects, as well as inhibitory effects on tumor cell invasion [18,19]. *E. angustifolia* has muscle-relaxing, anti-inflammatory, antinociceptive, antimutagenic properties [20,21,22], and inhibitory effects on memory impairment [23,24]. while *E. multiflora* has anti-inflammatory and antioxidative properties [25]. However, no studies have reported the therapeutic effects of EGFO, especially in models of AD and related cognitive disorders. Therefore, in this study, we aimed to determine whether EGFO can attenuate memory deficits in mice with scopolamine (SCO)-induced memory impairments, as well as the mechanisms underlying the effects of EGFO treatment. 

## 2. Materials and Methods

### 2.1. Preparation

*EGFO* was kindly provided by the Korean Seed Association. The botanical origin of this sample was taxonomically identified by Prof. Ju Hwan Kim of the Department of Life Sciences at Gacheon University. A voucher specimen was deposited at the Clinical Medicine Division of the Korea Institute of Oriental Medicine (SCD-A-112). Ethanol (2 × 30 L) extracts were obtained from dried EGFO branches (2.9 kg) via maceration using an electric extractor (COSMOS-660, Kyungseo Machine Co., Incheon, Korea) for 3 h at room temperature. Extracts were combined and concentrated *in vacuo* at 40 °C for lyophilization. The yield of the freeze-dried extract was 6.18%.

### 2.2. Experimental Animals and Drug Administration

All experiments were approved by the Korea Institute of Oriental Medicine Institutional Animal Care and Use Committee (IACUC Approval No.17-012) and performed in accordance with the National Institutes of Health (NIH) Guide for the Care and Use of Laboratory Animals. Male ICR mice were purchased at seven-week-old age (Daehan Biolink, Cheongju, Korea) and acclimated for one week prior to the study, with laboratory standard food and water provided *ad libitum* in individual cages. All mice were housed under controlled conditions (temperature: 21–23 °C, 12 h light/dark cycle, 55% humidity). Experiments were conducted using eight-week-old male ICR mice (weight: approximately 35 g), which were monitored for three weeks. A total of 50 male ICR mice were randomly divided to five groups; CONT: Normal control group, SCO: SCO-treated group, EGFO-50 or EGFO-100: EGFO (50 or 100 mg/kg)-treated SCO group, and a TAC: tacrine (10 mg/kg)-treated SCO group. Age-matched control mice administrated an equal volume of vehicle (phosphate buffered saline (PBS)). EGFO extracts were dissolved in distilled water to a concentration of 50 or 100 mg/kg and administered daily via gastric gavage. Tacrine (USP, Rockville, MD, USA) was used as a positive control. All mice underwent behavioral testing from days 14 to 19. Cognitive impairments were induced via a single injection of SCO (1 mg/kg, *i.p.*) within 60 min after oral administration of EGFO extract or tacrine. The behavioral tests were performed 30 min after SCO injection. The experimental design was modified based on the protocol of previous studies [26,27]. All efforts were made to minimize animal suffering. The animal equivalent dosage was determined from the human equivalent dose. We considered that the usual dosage of the plant is approximately 20–60g/60 kg/day of the raw plant for adult human. The calculated animal dose rang was from 40 to 120 mg/kg in mice. The EFGO dose for this study was determined 50 or 100 mg/kg per day. This animal study procedures were performed according to ARRIVE guideline.

### 2.3. Step-through Passive Avoidance Test

The passive avoidance test, conducted using two identical compartments (Gemini Avoidance system, San Diego, CA, USA), consisted of lighted and darkened compartment with an automated door in between compartments and electrifiable grid floor. During the acquisition phase, mice were placed in the lighted compartment for familiarization for 25 s and then crossed to the darkened compartment. The door was automatically opened and then received a mild electrical shock (0.3 mA, 3 sec). In retention phase, mice were again placed in the lighted compartment. The latency time of the darkened compartment required the mice to remain in lighted compartment was recorded as the retention time. If the mice did not enter the darkened compartment within 5 min, the latency time was recorded as 300 s. No physiological defects (i.e., motor deficits) or intrinsic cognitive impairments were observed in any of the mouse groups prior to treatment with scopolamine. 

### 2.4. Y-maze Test

The Y-maze (length: 35 cm; height: 15 cm; width: 7 cm) tests were positioned at equal angles three arms from one another. Each mouse was placed in one arm and allowed to freely explore the maze for 8 min. The number of spontaneous alternations was recorded using an Ethovision System (Noldus, Wageningen, Netherlands). Spontaneous alternation (%) was considered when mice entered into all three arms without repetition (e.g., ABC, BCA, and CAB). The rate of spontaneous alternation was calculated as follows:

% alternation = [(number of alternations)/(total number of arm entries − 2)] × 100.(1)

### 2.5. Brain Sectioning and Tissue Preparation

On day 21, all mice were deeply anesthetized (Traditional Anesthesia System, Torrington, CT, USA) via inhaled isoflurane (JW Pharmaceutical, Seoul, Korea) and sacrificed. Hippocampal and cortical tissues of seven mice were isolated and stored at −80 °C until analysis. The whole brains of three mice from each group were submerged in 4% paraformaldehyde (PAF) for tissue fixation.

### 2.6. Immunoblot Analysis

Hippocampal and cortical brain tissues were homogenized in RIPA buffer (pH: 7.5, Pierce Biotechnology, Rockford, IL, USA) with phosphatase and protease inhibitors. The proteins were separated on 4–20% gradient polyacrylamide gels and then transferred on PVDF membranes. Blocked PVDF membranes with 5% nonfat milk were washed with TBST and incubated using primary anti- phospho-CREB, nerve growth factor (NGF), ChAT, and cleaved caspase-3 (R&D system, Minneapolis, MN, USA) and Bax, Bcl2 (Santa Cruz Biotechnology, Santa Cruz, CA, USA). Washed membranes were incubated with secondary antibodies with conjugated horseradish peroxidase and immunoblot bands were developed using supersignal ECL solution (Amersham Bioscience, Piscataway, NJ, USA). Protein level were detected by analyzing the captured signals using a ChemiDox imaging analyzer (Las-4000 MINI, Fuji photo, Tokyo, Japan).

### 2.7. Determination of Ach Levels and AChE Activity in Brain Tissue

Ach levels and AChE activity in hippocampal and cortical tissues were measured using respective assay kits (US Biomax Inc, Denwood, MD, USA) in accordance with the manufacturer’s protocol. Absorbance of mixture was measured at 570 nm using a spectrophotometer (Benchmark Plus, Bio-Rad, Hercules, CA, USA).

### 2.8. Nissl and TUNEL Staining

Mouse brains were pre-fixed in 4% PFA and embedded with paraffin wax, following by sectioning to 4 μm thickness. The sections were deparaffinized and hydrated with xylene and an ethanol series (100%, 80%, 70%, and 50% ethanol). After rinsing in distilled water (DW), the slides were dipped in Nissl stain solution (0.5% cresyl violet) for 1 min and again rinsed with DW. TUNEL staining was performed using a TUNEL assay kit (Roche Diagnostics, Mannheim, Germany) in accordance with the manufacturer’s protocol. Apoptotic cells and fragmented DNA were evaluated using a purple color solution containing NBT/BCIP (nitroblue tetrazolium and 5-bromo-4-chloro-3-indolylphosphate, Roche Diagnostics). Stained sections were then visualized using an Olympus DP71 camera connected to an Olympus microscope (Tokyo, Japan) at 400×, 100×, or 10× magnification, respectively. The images were recorded and analyzed using Java-based image processing program Image J 1.52 software (NIH, Bethesda, MD, USA).

### 2.9. Statistical Analysis

All data were expressed as the mean ± SEM and statistical analyses were evaluated via a one-way analysis of variance (ANOVA) followed by an unpaired Student’s *t*-test or Tukey’s multiple comparison test. All experiments were performed individually at least three times. GraphPad *Prism 8.0* software (Graph pad, San Diego, CA, USA) was used for all analyses. Difference at *p* < 0.05 was considered to be statistical significance.

## 3. Results

### 3.1. Effect of EGFO in Mice with SCO-Induced Memory Impairments

**Figure 1:**
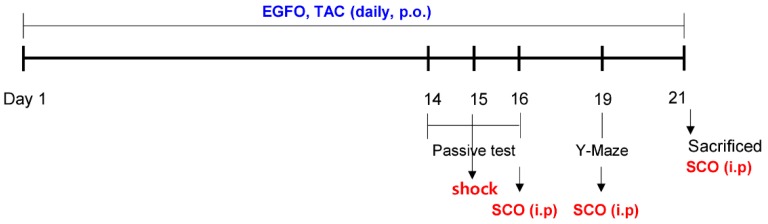
**Description of the experimental design**. ICR mice were orally administered vehicle, EGFO, or tacrine for 21 days. Scopolamine was administered 30 min prior to the step-through passive avoidance test (PAT) and Y-maze test. EGFO: *Elaeagnus glabra f. oxyphylla*; TAC: Tacrine.

**Figure 2:**
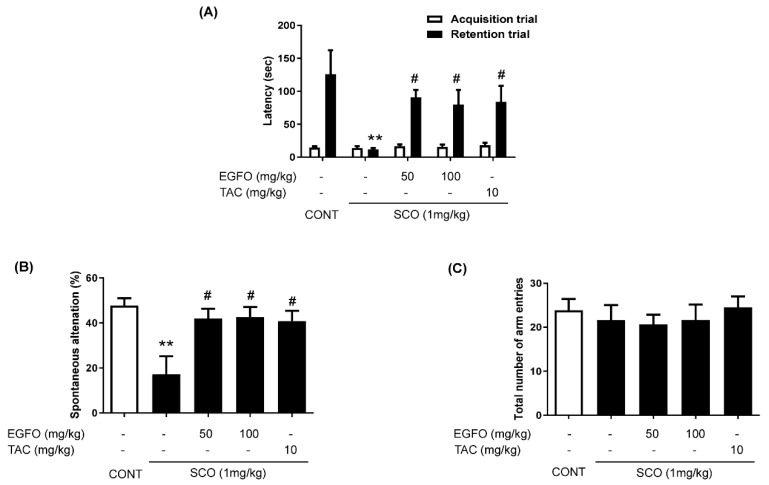
**Effect of EGFO extract on scopolamine-induced memory impairments**. (**A**) Twenty-four hours after the acquisition trials in the passive avoidance test (PAT), mice were subjected to 300-s retention trials. Spontaneous alternation (%) (**B**) and the total number of arm entries (**C**) were monitored for an 8-min session in the Y-maze test. The data are expressed as the mean ± SEM. (*n* = 10/group). ^**^ *p* < 0.01 vs. CONT group, ^#^ *p* < 0.01 vs. SCO group. CONT: Normal control; SCO: Scopolamine; EGFO: *Elaeagnus glabra f. oxyphylla*; TAC: Tacrine.

A schematic description of the animal experiments is presented in Figure 1. The body weight changes for baseline information revealed no marked differences between each group (Table 1). To determine whether EGFO promotes recovery of memory impairment, we performed step-through passive avoidance and Y-maze tests in mice with SCO-induced memory impairments. In the step-through passive avoidance test, we observed no significant differences in the latency to enter the darkened compartment among the groups during acquisition trials. Following the acquisition trials, we evaluated the effect of EGFO or tacrine (positive control [28,29]) on retention latency 24 h after applying electric shocks in the darkened compartment. Retention latency to enter the darkened compartment was significantly greater in the EGFO-50, EGFO-100, and TAC groups (*p* < 0.05) than in the SCO group (Figure 2A). 

We also evaluated the effects of EGFO on short-term memory function using the Y-maze test. As shown in Figure 2B,C, the rate of spontaneous alternation was significantly decreased in the SCO group, although this effect was significantly attenuated by treatment with EGFO at 50 mg/kg or 100 mg/kg, respectively. Tacrine treatment also attenuated SCO-induced memory deficits. No significant differences in the total number of arm entries were observed among the groups, suggesting that SCO, EGFO, and tacrine did not affect locomotor activity. 

### 3.2. Effect of EGFO Extract on Cholinergic Dysfunction in Mice with SCO-Induced Memory Impairments

**Figure 3:**
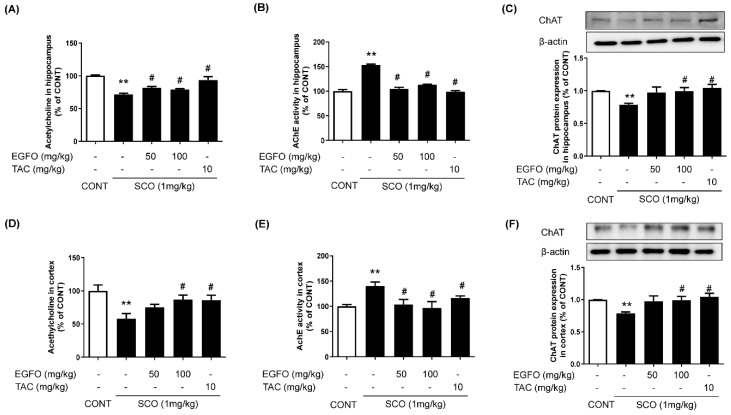
**Effect of EGFO extract on cholinergic dysfunction in mice with scopolamine-induced memory impairments**. Ach levels (**A**,**D**) and AChE activity (**B**,**E**) in the hippocampus (**A**,**B**) and cortex (**D**,**E**) were measured using respective assay kits (US Biomax Inc., Denwood, MD, USA). (**C**,**F**) Hippocampal and cortical tissues were lysed and subjected to Western blotting using an anti-ChAT antibody. Levels of expression were normalized by β-actin. Bar graphs represent the relative intensity in each band. Values are expressed as the mean ± SEM. (*n* = 7/group). ^**^ *p* < 0.01 vs CONT group, ^#^ *p* < 0.01 vs. SCO group. Ach: Acetylcholine; AChE: Acetylcholinesterase; ChAT: Choline acetyltransferase; CONT: Normal control; SCO: Scopolamine; EGFO: *Elaeagnus glabra f. oxyphylla*; TAC: Tacrine.

A major excitatory neurotransmitter, Ach, is critical for memory formation and retention [28]. Thus, we measured cholinergic indices to investigate the potential effects of EGFO on memory loss in SCO-treated mice. The SCO group exhibited a remarkable decrease in Ach levels (left column) and increased AChE activity (middle column) in both the hippocampus and cortex. In contrast, EGFO or tacrine treatment significantly attenuated the effects of SCO on Ach levels (left column) and AChE activity (middle column). In addition, the SCO group exhibited significant decreases in ChAT protein expression in the hippocampus and cortex, which were also attenuated by EGFO or tacrine treatment (Figure 3C,F). These results indicate that EGFO may protect against SCO-induced dysfunction of the cholinergic system in the brain. 

### 3.3. Effect of EGFO Extract on the Neuronal Markers CREB and NGF in Mice with SCO-Induced Memory Impairments

**Figure 4:**
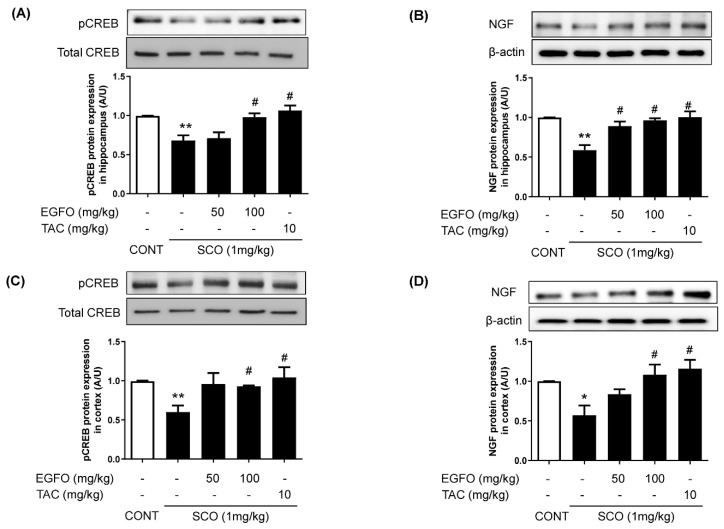
**Effect of EGFO extract on phospho-CREB and NGF levels in mice with scopolamine-induced memory impairments**. Hippocampal and cortical tissues were homogenized and performed Western blotting with anti-phospho-CREB or NGF antibodies. Levels of phosphor-CREB **(A**,**C**) and NGF (**B**,**D**) expression in hippocampus and cortex were normalized by total CREB or β-actin, respectively. Bar graphs represent the relative intensity in each band. Values are expressed as the mean ± SEM. (*n* = 7/group). ^*^ *p* < 0.05, ^**^ *p* < 0.01 vs. CONT group, ^#^ *p* < 0.05 vs. SCO group. CREB: cAMP response element-binding protein; NGF: Nerve growth factor; CONT: Normal control; SCO: Scopolamine; EGFO: *Elaeagnus glabra f. oxyphylla*; TAC: Tacrine.

CREB plays a critical role in neuronal survival, growth, proliferation, and differentiation [30]. NGF is a central neurotrophin that modulates memory and participates in the CREB phosphorylation [31,32]. Thus, we examined the effect of EGFO extract on levels of CREB phosphorylation and NGF expression in both the hippocampus and cortex of mice brain with SCO-induced memory impairments. CREB phosphorylation and NGF expression were significantly lower in the SCO group than in the CONT group. However, EGFO treatment significantly attenuated the suppressive effects of SCO on CREB and NGF (*p* < 0.05) (Figure 4A,B). Tacrine treatment exerted effects similar to those of EGFO on CREB and NGF levels. 

### 3.4. Protective Effect of EGFO Extract against Neuronal Damage in Mice with SCO-Induced Memory Impairments

**Figure 5:**
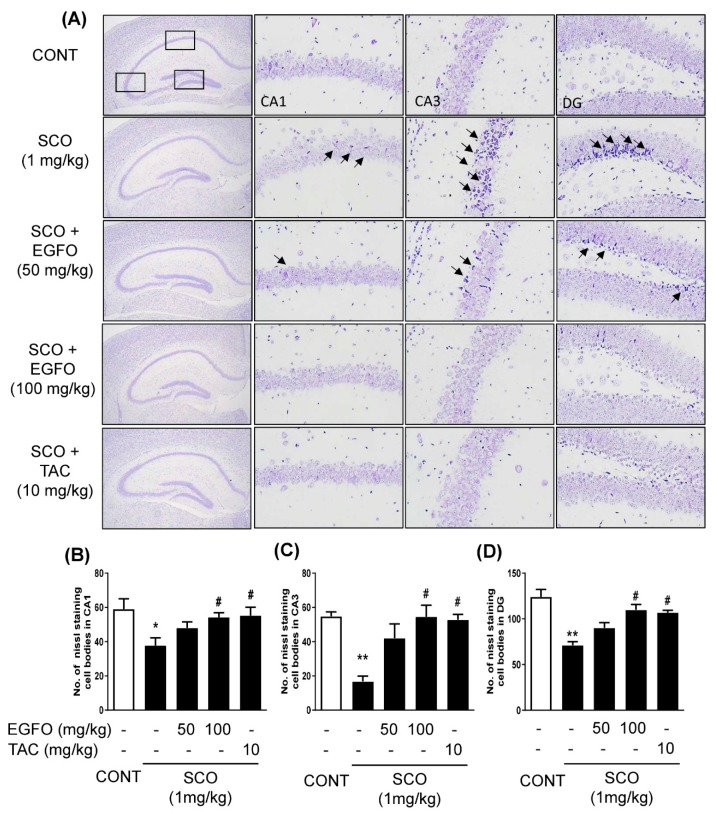
**Effect of EGFO extract on morphological damage to neurons in mice with scopolamine-induced memory impairments**. Sections of the hippocampal CA1, CA3, and dentate gyrus (DG) were regions were stained with Nissl staining solution. Representative photomicrographs (**A**) are shown at magnifications of 40× (first column) and 400× (from second to fourth column). Bar graphs represent the number of Nissl staining cell bodies in histological sections of the CA1 (**B**), CA3 (**C**), and DG (**D**). The statistic difference indicated such as * *p* < 0.05, ^**^ *p* < 0.01 vs. CONT group, or ^#^ *p* < 0.05 vs. SCO group (*n* = 3/group). CONT: Normal control, SCO: Scopolamine, EGFO: *Elaeagnus glabra f. oxyphylla*; TAC: Tacrine; CA: Cornu ammonis.

Changes in neuronal cell morphology were identified based on cresyl violet staining in the CA1, CA3, and DG regions of hippocampal tissue. Healthy neuronal cells were observed in the CONT group. However, the SCO group exhibited marked neuronal damage, nucleus shrinkage, and altered staining (arrows) in the CA1, CA3, and DG areas of the hippocampal region compared to the CONT group. Treatment with EGFO or tacrine attenuated the effects of SCO on neurons in the hippocampal region (Figure 5). 

### 3.5. Effects of EGFO Extract on the Apoptosis Pathway in Mice with SCO-Induced Memory Impairments

**Figure 6:**
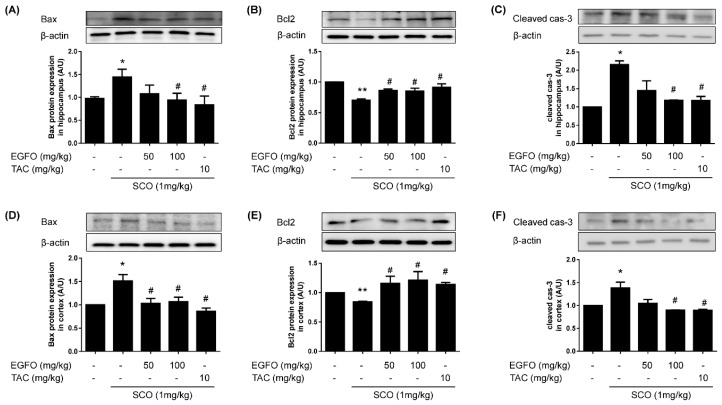
**Effect of EGFO extract on the expression of apoptosis-related proteins in mice with scopolamine-induced memory impairments**. Lysed hippocampal and cortical tissues performed Western blotting with anti-Bax (**A**,**D**), Bcl2 (**B**,**E**), and cleaved caspase-3 (**C**,**F**) antibodies. Levels of expression were normalized to β-actin. Bar graphs represent the relative intensity in each band. Values are expressed as the mean ± SEM. (*n* = 7/group). ^*^ *p* < 0.05, ^**^ *p* < 0.01 vs CONT group, ^#^ *p* < 0.05 vs. SCO group. CONT: Normal control; SCO: Scopolamine; EGFO: *Elaeagnus glabra f. oxyphylla;* TAC: Tacrine.

To determine whether EGFO treatment attenuates SCO-induced apoptosis, we performed Western blotting assays using antibodies for apoptosis-related proteins such as Bcl2, Bax, and cleaved caspase-3. As shown in Figure 6, SCO treatment significantly increased levels of Bax and cleaved caspase-3 while decreasing Bcl2 expression in both the hippocampus and cortex. However, such changes were significantly attenuated by EGFO or tacrine treatment. 

**Figure 7:**
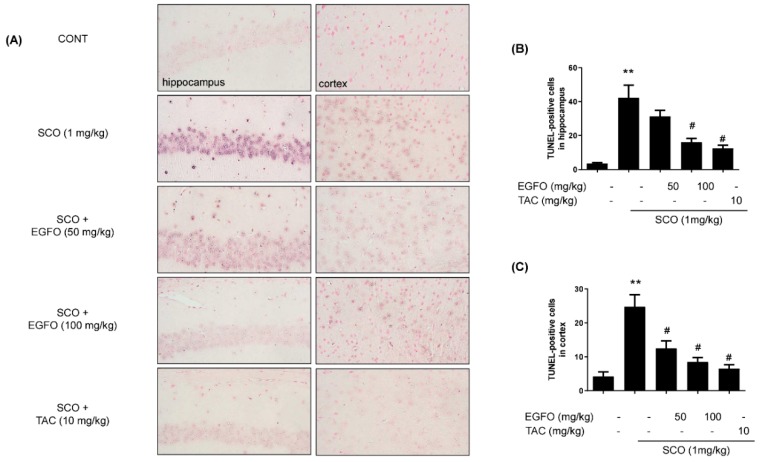
**Effect of EGFO extract on apoptosis in mice with scopolamine-induced memory impairments.** (**A**) Representative photomicrographs were obtained at a magnification of 400×. Apoptotic cells were visualized using nitroblue tetrazolium and 5-bromo-4-chloro-3-indolylphosphate (NBT/BCIP-AP, purple). Counterstaining for the nucleus was performed using Fast-red. (**B**,**C**). Bar graphs represent the quantitative results for apoptotic signals in histological sections of the hippocampus (**B**) and cortex (**C**). Experimental values are expressed as the mean ± SEM. (*n* = 3/group). ^**^ *p* < 0.01 vs. CONT group, ^#^ *p* < 0.05 vs. SCO group. CONT: Normal control; SCO: Scopolamine; EGFO: *Elaeagnus glabra f. oxyphylla*; TAC: Tacrine.

TUNEL assays were performed to further verify the inhibitory effect of EGFO against SCO-induced neuronal apoptosis. In the CONT group, TUNEL-labeled cells were rarely shown in the hippocampus and cortex. However, the SCO group exhibited prominent increases in the number of TUNEL-labeled cells in both the hippocampus and cortex when compared with the CONT group. In contrast, EGFO or tacrine treatment significantly attenuated neuronal apoptosis in these regions in SCO-treated mice (Figure 7).

## 4. Discussion

Neurodegenerative diseases such as AD represent a notable healthcare issue, as they lead to progressive brain dysfunction as well as learning and memory impairments among older adults. In the present study, we investigated whether EGFO can improve memory function in mice with SCO-induced memory impairments, which are similar to those observed in patients with such neurodegenerative conditions.

Cholinergic dysfunction is among the leading causes of impairments in memory processing in patients with AD [33]. SCO, a cholinergic blocking agent, is commonly used to develop animal models of AD/memory impairment, inducing what is referred to as “scopolamine dementia” [25,34]. SCO treatment results in degenerative changes within the forebrain cholinergic system, producing severe deficits in learning and memory function as assessed using inhibitory avoidance and spatial learning tasks [25,35]. In the present study, we utilized the passive avoidance and Y-maze tests to assess fear-motivated memory retrieval [36] and spatial learning/memory in mice, respectively [37]. Results on these behavioral tests have been associated with damage to the cholinergic system in areas of the basal forebrain such as the hippocampus and cortex [38,39,40]. Ach, which is hydrolyzed by AChE, is well-known enzyme which plays a pivotal role in learning and memory [41]. ChAT is a critical cholinergic marker participated in Ach synthesis [42]. Maintenance of Ach is essential for normal function, whereas inordinate AChE activity result in disruptions in Ach levels in the brain [43]. Consistent with previous findings, our results demonstrated that SCO significantly decreased spontaneous alternation and retention time in mice, while EGFO treatment significantly attenuated these SCO-induced memory impairments. In addition, SCO treatment significantly decreased Ach levels and expression of ChAT protein while increasing AChE activity in both the hippocampus and cortex, suggesting that the observed cognitive impairments were induced by cholinergic dysfunction. However, EGFO treatment markedly attenuated SCO-mediated cholinergic dysfunction, exerting effects similar to the cholinesterase inhibitor tacrine [29,44], which is also known to enhance memory function. These results suggest that EGFO enhances memory function in mice with SCO-induced memory impairments via anticholinergic mechanisms. 

NGF, the first neurotrophin discovered, plays a pivotal role in neuronal plasticity and neurogenesis via the inhibition of CREB phosphorylation [45,46]. Impaired CREB phosphorylation is a known pathological factor of neurodegenerative disorders [47], triggering neuronal loss in the hippocampus and cortex via a pro-apoptotic process [48,49,50]. Inhibition of CREB impairs behavioral performance on various memory tests. In contrast, overexpression of CREB promotes neuronal survival [51,52] and ameliorates cognitive impairments via the cholinergic system [53]. In the present study, SCO treatment significantly reduced CREB phosphorylation and NGF expression in the hippocampus and cortex. In addition, SCO enhanced Bax expression and caspase-3 cleavage, and reduced Bcl-2 expression when compared with levels observed in the NC group. Furthermore, TUNEL staining experiments indicated that SCO significantly increased neuronal apoptosis in these two brain regions, although these effects were attenuated by EGFO treatment. Taken together, these results indicate that EGFO may ameliorate learning and memory impairments by activating neurotrophic factors and preventing neuronal apoptosis. 

To date, reversible cholinesterase inhibitors such as tacrine, donepezil, and rivastigmine have been used in the treatment of mild to moderate AD. However, due to their low efficacy and numerous adverse effects, the therapeutic potential of these drugs is limited [54]. Therefore, researchers have expressed increasing interest in cholinesterase inhibitors derived from natural products. To explore the toxicological feature of EGFO, we performed the acute toxicity test and found no toxicity of EGFO at doses up to 5,000 mg/kg in SD rats (data not shown). The dose of acute toxicity in rat was calculated at 10,000 mg/kg by equivalent surface area conversion in mice. Animal experiments were performed under nontoxic concentration of EGFO. Indeed, several studies have suggested that natural products or medicinal herbs such as pine needle [55], *Actinidia argute* [56], and *Avena sativa* [57] can enhance memory in both animals and humans with fewer adverse effects than current pharmacological agents.

In general, the efficacy of natural product extracts may be enhanced by advantages of synergy and interactions among the various phytochemicals. We are working to determine the standard compounds to establish quality control of EGFO using high-performance liquid chromatography analysis. After identifying the standard compounds composing EGFO, the potential bioactive compound of EGFO and its blood-brain barrier permeability will be determined in the near future. 

## 5. Conclusions

Our results demonstrated the memory-enhancing effects of the natural product EGFO in mice with SCO-induced memory impairments. Further analysis revealed that EGFO exerted protective effects on the cholinergic system and promoted neurogenesis by activating NGF/CREB signaling and inhibiting apoptosis-related pathways. Overall, our findings may aid in the development of novel pharmacological agents for the treatment of learning and memory impairments in patients with AD and related neurodegenerative diseases.

## Figures and Tables

**Table 1:** Experimental body weight changes.

Body Weight (g)	CONT	SCO	SCO + EGFO-50	SCO + EGFO-100	SCO + TAC
Initial	35.35 ± 0.32	34.54 ± 0.60	35.66 ± 0.31	34.21 ± 0.69	35.39 ± 0.54
End	37.66 ± 0.60	37.32 ± 0.73	37.00 ± 0.52	36.05 ± 0.62	36.59 ± 0.67

CONT: Normal control; SCO: Scopolamine; EGFO: *Elaeagnus glabra f. oxyphylla*; TAC: Tacrine. The data are expressed as the mean ± SEM. (*n* = 10/group).
